# Grading systems and perineural invasion in oral squamous cell carcinoma - a disease-specific survival analysis

**DOI:** 10.4317/medoral.26896

**Published:** 2025-01-26

**Authors:** Sibele Nascimento de Aquino, Lucas Lacerda de Souza, Daniel Alvarenga, Paulo Rogério Ferreti Bonan, Helder Domiciano Dantas Martins, Francielle Silvestre Verner, Márcio Ajudarte Lopes, Pablo Agustin Vargas

**Affiliations:** 1 Applied Health Sciences Post-Graduate Program, Federal University of Juiz de Fora, Governador Valadares, Minas Gerais, Brazil; 2 Department of Oral Diagnosis, Piracicaba Dental School, University of Campinas, Piracicaba, Brazil; 3 Department of Medicine, Federal University of Juiz de Fora, Governador Valadares, Minas Gerais, Brazil; 4 Post-Graduate Program in Dentistry, Federal University of Paraíba, João Pessoa, Brazil

## Abstract

**Background:**

Oral squamous cell carcinoma (OSCC) is an aggressive cancer, with prognosis influenced by clinical variables as well grading systems and perineural invasion (PNI), which are associated to poorer outcomes, including higher rates of recurrence and metastasis. This study aims to evaluate OSCC using three grading systems and assess the impact of PNI and clinicopathologic parameters on patient survival.

**Material and Methods:**

Eighty-one primary OSCC samples were analyzed. Histopathological evaluations were performed utilizing Malignancy Grading of the Deep Invasive Margins, WHO grading system, and the Histologic Risk Assessment. S-100 immunohistochemistry was used to detect PNI. Five-year disease-specific survival (DSS) curves were generated using the Kaplan-Meier method, and the Cox proportional hazards model analyzed prognostic significance.

**Results:**

Advanced clinical stage was significantly associated with reduced survival (*p-value* <0.001, HR = 4.07). Patients without regional lymph node involvement had better survival (*p-value* 0.002, HR = 0.37). Higher histologic risk assessment scores were linked to worse outcomes. Multifocal neural invasion significantly correlated with poorer survival compared to unifocal invasion (*p-value* 0.017, HR = 4.20). Patients undergoing surgery followed by adjuvant therapies had better survival rates.

**Conclusions:**

Besides clinical stage and histological grade, PNI also showed to be a crucial prognostic factor in OSCC, necessitating aggressive treatment strategies.

** Key words:**Squamous cell carcinoma, histological grade, neural invasion, prognosis, outcome.

## Introduction

Oral squamous cell carcinoma (OSCC) is the most prevalent type of oral cancer, predominantly associated to chronic consumption of tobacco, alcohol, and betel nut, varying by geographical region (1). Surgery is the most common treatment modality, with radiation and/or chemotherapy often used as adjuvant therapy (2). Typically, treatment decisions are guided by the TNM classification system (3). However, even with identical treatment plans, patients at the same TNM stage can experience different outcomes. To address this variability, researchers have developed various histological grading systems to enhance the TNM staging system and improve the prediction of clinical outcomes, offering greater prognostic accuracy (4-8).

In 1920, Broders introduced a classification system for lip cancer, based on the resemblance of the neoplasm to normal squamous epithelium (4). The Multiparameter Grading System, established in 1986 by Anneroth *et al*., evaluated key histopathologic features such as keratinization, nuclear pleomorphism, mitotic activity, and the tumor-host relationship, including the pattern and depth of invasion and inflammatory infiltration (5). In 1992, Bryne *et al*. (6) proposed the Malignancy Grading of the Deep Invasive Margins for OSCC, focusing on the most invasive tumor cell front at the tumor-host interface.

The World Health Organization (WHO) revised its grading system in 2005 to categorize squamous cell carcinomas based on the degree of cell differentiation, dividing them into well-differentiated, moderately differentiated, and poorly differentiated tumors (7). Additionally, in 2005, Brandwein-Gensler *et al*. introduced the Histologic Risk Assessment, which evaluates the worst pattern of invasion, perineural invasion (PNI), and the lymphocytic response to tumors. This system assigns different scores for each feature, classifying patients into low-, intermediate-, and high-risk groups based on their likelihood of local recurrence and overall survival probability (8).

The evaluation of neural invasion was integrated into the grading system proposed by Brandwein-Gensler *et al*. (8) due to the neurotropic nature of OSCC, which can affect peripheral nerves in the head and neck region (9). PNI has been associated to adverse outcomes, including lymph node metastasis, locoregional recurrence, and poor overall, disease-free, and disease-specific survival (10,11). In the Histologic Risk Assessment grading system, PNI is classified into three categories: none, involvement of small nerves (<1 mm), or involvement of large nerves (>1 mm), assigned scores of 0, 1, and 3, respectively (12). However, some researchers have argued that further subcategorization of neural involvement—such as distinguishing between unifocal and multifocal, and between intratumoral and peripheral—could improve prognostic predictions (13-15).

While the prognostic value of PNI is increasingly recognized (9,11,14,15), its effectiveness in prediction models that integrate other histopathological factors and grading systems remains underexplored. Therefore, the aim of this study was to examine the Malignancy Grading of the Deep Invasive Margins (6), the WHO grading system (7), and the Histologic Risk Assessment (8), along with the subcategorization of PNI in primary OSCC. This comprehensive approach seeks to understand their collective impact on prognostic factors and patient survival.

## Material and Methods

- Study design

Cases diagnosed as OSCC were retrospectively from two Pathological Anatomy centres. Formalin-fixed paraffin-embedded (FFPE) tissue blocks were obtained, and new 4μm histological sections were cut and stained with hematoxylin and eosin (H&E) for microscopic description and diagnostic confirmation following the 5th edition of the World Health Organization Classification of Head and Neck Tumors (16). We included patients who had only undergone surgical resections for previously untreated tumors. We excluded cases that lacked a complete clinical history or had insufficient tissue in the FFPE block. Cases diagnosed in the oropharyngeal region were also excluded.

The following information were extracted from the clinical records: patient sex, age at diagnosis, history of alcohol and/or tobacco consumption, tumor location, TNM classification according to the AJCC 8th edition (17), treatment (surgery alone or with radiotherapy and/or adjuvant chemotherapy), date of diagnosis, date of surgery, date and cause of death. Only patients with a minimum of 5 years of follow-up or who were deceased because of the disease were included.

- Histopathological analysis

Hematoxylin and eosin staining was conducted on all cases, and the slides were graded according to three established systems: the Malignancy Grading of the Deep Invasive Margins (6), the WHO grading system (7), and the Histologic Risk Assessment (8). For the Malignancy Grading of the Deep Invasive Margins, we assessed tumor cell characteristics such as the degree of keratinization, nuclear polymorphism, and the number of mitoses, as well as the tumor-host relationship, including the pattern and stage of invasion and lymphoplasmacytic infiltration. Each histopathologic feature was assigned a score from 1 to 4 (6), which were then summed to estimate the overall malignancy of the tumor. Using the WHO grading system, tumors were categorized into three levels of differentiation: well-differentiated, moderately differentiated, and poorly differentiated (7). The Histologic Risk Assessment involved evaluating lymphocytic infiltrate, the worst pattern of invasion, and perineural invasion (8). Based on these evaluations, tumors were further classified as Grade I, II, or III.

- Immunohistochemistry

Immunohistochemistry (IHC) was conducted on 3-μm-thick sections of formalin-fixed, paraffin-embedded tissue samples. Primary antibody targeting the S-100 protein (Dako, Carpinteria, CA, dilution 1:10000) was used to evaluate the presence of perineural invasion. The tissue sections were deparaffinized and hydrated before undergoing antigen retrieval, using either an EDTA/Tris solution (pH 9.0) or a citric acid solution (pH 6.0), as recommended by the manufacturer, for 15 minutes in an electric pressure cooker. Endogenous peroxidase activity was blocked with 6% hydrogen peroxide for 15 minutes. After this pretreatment, the sections were incubated with the primary antibody for 2 hours at room temperature. For detection, the Envision-Dual Link System-HRP (Dako, Carpinteria, CA) was applied according to the manufacturer’s protocol, and staining was performed with diaminobenzidine (Dako, Carpinteria, CA) for 5 minutes. The sections were then counterstained with Carazzi’s hematoxylin for 3 minutes. Positive controls were included, while negative controls were prepared by omitting the primary antibody.

The size of the largest involved nerve was classified as major or minor based on the diameter of the nerve bundle (greater or less than 1 mm, respectively) (13). The extent of perineural invasion (PNI) was recorded as unifocal or multifocal, depending on whether a single focus or multiple foci of PNI were observed, respectively (14).

- Statistical analysis

K-means clustering was employed to determine cutoff points for the categories, aiming to dichotomize them. The collected data were summarized using crosstabs, and associations between variables were evaluated using the chi-square test or Fisher's exact test. The focus of the survival analysis was on 5-year disease-specific survival (DSS), which is defined as the time between the date of diagnosis and the date of death due to OSCC. Survival curves were created using the Kaplan-Meier method and compared with the log-rank test. To identify independent risk factors associated with poor survival, the Cox proportional hazard model was used. Backward elimination was utilized to select the covariates included in the regression models. Data processing was carried out using JAMOVI (by the JAMOVI Project) software. The level of statistical significance was set at 5% (*P* ≤ 0.05).

## Results

- Demographic characteristics

A total of 81 cases diagnosed as OSCC were retrieved from the assesses pathology files and included in the current study for analysis.

Table 1 presents the clinicopathologic features of the study cohort. Among the 81 patients included, 16 (19.75%) were under 50 years of age, while 65 (80.24%) were 50 years or older. The cohort consisted of 59 men (72.83%) and 22 women (27.16%). The primary tumor sites were the tongue in 38 patients (46.91%), the floor of the mouth in 19 patients (23.45%), the hard palate in 9 patients (11.1%), the buccal mucosa in 3 patients (3.70%), the retromolar region in 4 patients (4.93%), and multiple sites in 8 patients (9.87%). Smoking status showed that 17 patients were non-smokers (20.98%) and 52 were smokers (64.19%). Drinking status evidenced 29 individuals were never-Drinkers (35.80%), while 35 were drinkers (43.20%). Tumor stages were grouped into T1-2 (32 patients, 39.50%) and T3-4 (46 patients, 56.79%). Regional lymph node involvement was assessed, and 40 patients (49.38%) presented no lymph node involvement, while 38 patients (46.91%) showed regional lymph node involvement.

Tumor size revealed that 32 patients (39.5%) were in the early stages (1-2), while 46 (56.79%) were in the more advanced stages (3-4). Regional lymph node involvement was observed in 38 patients (46.91%), whereas 40 patients (49.38%) showed no lymph node involvement. Clinical stage showed 22 patients (27.16%) were classified as stages I-II, and 58 (71.60%) were classified as stages III-IV.

The treatment modalities varied among the patients. Radiotherapy was administered to 52 patients (64.19%), while 19 (23.45%) did not receive this model of treatment. Chemotherapy was used as treatment modality in 34 patients (41.97%), while in 33 patients (40.74%) it was not used. Additionally, 24 patients (29.62%) received a combination of surgery and either radiotherapy or chemotherapy.

- Histopathologic classification analysis

Histologic description

The histopathological assessment, based on Broders' classification and described by the WHO in 2005, showed no significant association with tumor size, lymph node metastasis, and clinical stage, similarly to the Malignancy Grading of Deep Invasive margins, described by Bryne *et al*. in 1992. When considering the histologic risk assessment described by Brandwein-Gensler in 2005, we found an association with tumor size (P-value: ≤0.001) and clinical stage (P-value: ≤0.001), and a tendency towards significance in lymph node metastasis (P-value: 0.05). In terms of neural invasion (as indicated by S100 expression), tumor size (P-value: 0.31) and lymph node metastasis (P-value: 0.73) did not show any significant results, but there was a significant result for clinical stage (P-value: ≤0.006). When evaluating the foci of neural invasion, clinical stage evidenced significant result (P-value: 0.01). (Table 2).

- Survival Analysis

The univariate survival analysis showed that the absence of regional lymph node involvement was associated with improved survival (P-value: 0.002, HR = 0.37, 95% CI: 0.20-0.69) when compared to the presence of lymph node involvement. Clinical stage also demonstrated a significant relationship, with stages III-IV associated with reduced survival (P-value: 0.015, HR = 2.60, 95% CI: 1.20-5.59) relative to stages I-II. Furthermore, the analysis indicated that patients who did not undergo surgery with CT or RT had significantly reduced survival (P-value: 0.016, HR = 2.41, 95% CI: 1.18-4.93) compared to those who received these treatments.

**Figure 1:**
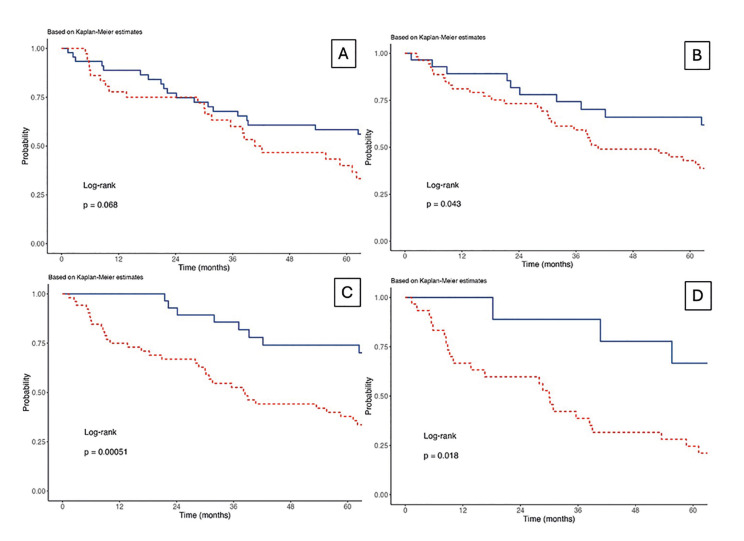
A - WHO/Broders (2005) (Well-differentiaded x moderately/poorly differentiated); B - Malignancy grading of deep invasive margins (≤8 points x ≥9 points); C - Histologic risk assessment by Brandwein-Gensler *et al*. (2005) (≤2 points x ≥3 points); D - Neural Invasion - Foci (unifocal x multifocal).

**Figure 2:**
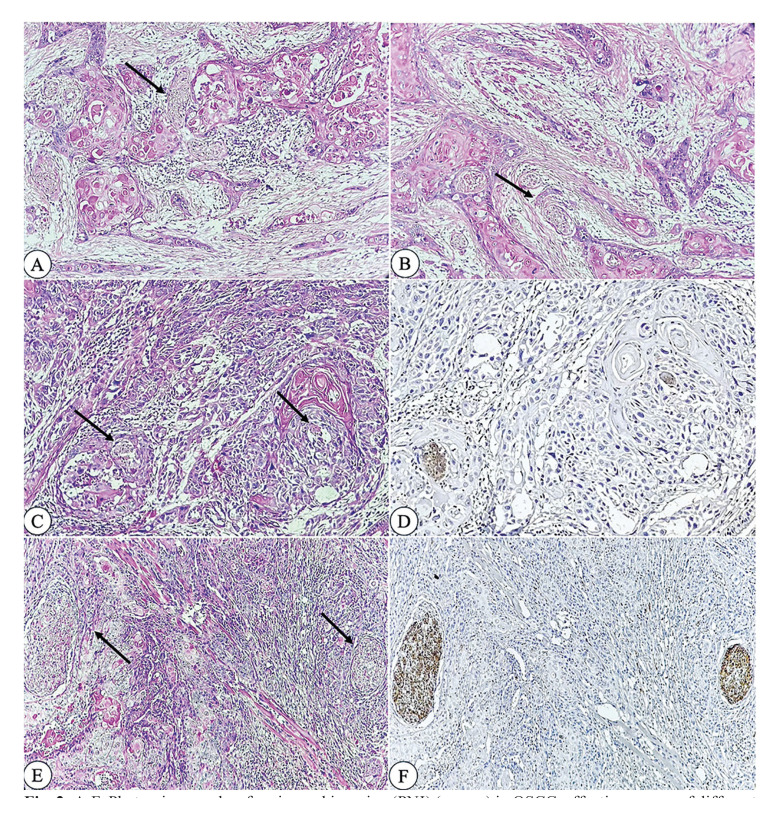
A-F: Photomicrographs of perineural invasion (PNI) (arrows) in OSCC, affecting nerves of different sizes as well multifocal involvement. A and B - PNI in different regions of the same tumor (H&E, 10x). C- Presence of PNI in adjacent regions in the same tumor, involving minor nerve (H&E, 10x). D - Same region as in photomicrographs C, with S100 staining (10x). E and F: Another case of multifocal PNI, involving a major nerve (H&E and S100, 4x, respectively).

The malignancy grading of deep invasive margins, as defined by Bryne *et al*. (1992), showed that patients with ≥9 points had significantly reduced survival (P-value: 0.047, HR = 1.98, 95% CI: 1.01-3.90) compared to those with ≤8 points. The histologic risk assessment by Brandwein-Gensler *et al*. (2005) revealed that patients with a score of ≥3 points had significantly reduced survival (P-value: 0.001, HR = 3.26, 95% CI: 1.61-6.59) compared to those with 0-2 points. Additionally, multifocal neural invasion was associated with significantly reduced survival (P-value: 0.025, HR = 3.38, 95% CI: 1.16-9.83) compared to unifocal invasion (Fig. 1). Fig. 2 illustrates the presence of PNI.

Radiotherapy approached statistical significance for patients not receiving it (P-value: 0.054, HR = 1.92, 95% CI: 0.99-3.73) compared to those receiving it. Chemotherapy did not demonstrate statistical significance, with patients not receiving it (P-value: 0.941, HR = 0.98, 95% CI: 0.52-1.84) compared to those who did. Lastly, the WHO - Broders (2005) classification approached statistical significance for moderately/poorly differentiated tumors (P-value: 0.071, HR = 1.70, 95% CI: 0.96-3.02) compared to well-differentiated tumors.

The multivariate analysis revealed that the presence of lymph node involvement was associated with significantly reduced survival (P-value <0.001, HR = 2.45, 95% CI: 1.89-5.97) compared to the absence of lymph node involvement. Clinical stage also had a significant impact, with stages III-IV associated with reduced survival (P-value <0.001, HR = 4.07, 95% CI: 1.21-13.63) relative to stages I-II. Furthermore, the analysis indicated that patients who did not undergo surgery with CT or RT had significantly reduced survival (P-value <0.001, HR = 2.72, 95% CI: 1.04-7.29) compared to those who received these treatments.

The Malignancy Grading of the Deep Invasive Margins by Bryne *et al*. (1992) did not show significant differences in survival, with a hazard ratio of 1.00 (P-value 1.0, 95% CI: 0.10-10.0) for patients with ≥9 points compared to those with ≤8 points. The Histologic Risk Assessment by Brandwein-Gensler *et al*. (2005) showed that patients with a score of ≥3 points had a hazard ratio of 16.36 (P-value 0.761, 95% CI: 0.16-12.61), indicating no significant difference in survival compared to those with 0-2 points. Lastly, multifocal neural invasion was associated with reduced survival (P-value 0.017, HR = 4.20, 95% CI: 1.29-13.66) compared to unifocal invasion (Table 3).

## Discussion

PNI is increasingly recognized as an important prognostic indicator in OSCC, suggesting a potential association with aggressive tumor behavior (10). PNI is associated with an increased likelihood of local and regional recurrence, as well as distant metastasis, which collectively contribute to poorer overall survival rates (10,18). The presence of PNI in OSCC patients necessitates more aggressive treatment strategies, including adjuvant therapies such as radiotherapy or chemoradiotherapy (19). Our study corroborates these observations, demonstrating that multifocal neural invasion is significantly linked to reduced survival compared to unifocal invasion, thereby underscoring the prognostic importance of PNI in clinical practice (20).

The majority of patients in our study were aged 50 years or older, with a higher prevalence among males, aligning with demographic trends observed in OSCC (21). The tongue was identified as the most common primary tumor site, followed by the floor of the mouth, which reflects the high anatomical density of locoregional nerves in these areas (19-21). Smoking and alcohol consumption were prevalent lifestyle habits among the patients, both of which are known risk factors for OSCC development (21). Our findings revealed that a significant proportion of patients were diagnosed at advanced tumor stages (III and IV), with nearly half exhibiting regional lymph node involvement. These characteristics are well-documented predictors of poor prognosis in OSCC (22).

The data also demonstrated a significant association between advanced clinical stage and reduced survival, emphasizing the critical importance of early detection and comprehensive staging in improving patient outcomes (22,23). In addition, our study found that patients who underwent surgery followed by adjuvant radiotherapy or chemotherapy had better survival outcomes. This could be attributed to the comprehensive treatment approach required for patients with advanced disease stages, which necessitates both surgical intervention and adjuvant therapies to effectively manage the disease. This approach is supported by existing literature, which highlights the importance of combining surgery with additional treatments to improve prognosis in patients with advanced stages of the disease (14,16,18).

The histologic risk assessment by Brandwein-Gensler and colleagues has emerged as a significant predictor of survival in our study, with higher scores correlating with poorer outcomes. This finding underscores the importance of detailed histologic evaluations, including the assessment of PNI, in prognostication and treatment planning (8,24). Our results align with those reported by Brandwein-Gensler *et al*. (8), who also highlighted the adverse impact of PNI on survival. Specifically, multifocal neural invasion was identified as an independent prognostic factor, reinforcing the need for meticulous assessment of PNI during histopathological examination. Although the similarities with Brandwein-Gensler *et al*. (8), our study shows variations in sample size, cohort characteristics, and the specific variables included. While we aimed to align with Brandwein-Gensler’s findings, the inherent differences in the patient populations, histopathological analysis and treatment protocols could explain why our results did not reach statistical significance in other variables in the multivariate analysis. In addition to the present result, our research evidenced multifocal PNI, which is consistent with previous studies that underscore the significance of PNI in OSCC, including the presence of multifocal invasion (9,10,11,14,15).

Moreover, research by Spoerl *et al*. (10) demonstrated that PNI correlates with increased cumulative and distant metastasis rates, further highlighting its role as an adverse prognostic feature. The inclusion of PNI in staging and risk assessments can significantly inform clinical decisions, particularly concerning the need for elective neck dissection and adjuvant therapies. These comprehensive evaluations and treatment strategies are critical in improving the management and outcomes of patients with OSCC (18,20,22,23).

## Conclusions

The histologic risk assessment by Brandwein-Gensler *et al*. has been validated as a predictor of survival, reinforcing the necessity for detailed histopathological evaluations, including meticulous PNI assessment. PNI is a critical prognostic indicator in OSCC, reflecting aggressive tumor behavior and associated with poorer overall survival rates due to increased local, regional, and distant recurrence. The need of incorporating adjuvant therapies such as radiotherapy or chemoradiotherapy in treatment strategies for patients with PNI is evident, as supported by our findings that multifocal neural invasion significantly reduces survival compared to unifocal invasion. This cohort emphasizes the importance of early detection and comprehensive staging to improve patient outcomes.

## Figures and Tables

**Table 1:** Clinicopathologic findings of the analyzed cases.

Variable		N (%)
Age	< 50 years	16 (19.75)
	> 50 years	65 (80.24)
Sex	Male	59 (72.83)
	Female	22 (27.16)
Tumor site	Tongue	38 (46.91)
	Floor of the mouth	19 (23.45)
	Hard palate	9 (11.1)
	Buccal mucosa	3 (3.70)
	Retromolar region	4 (4.93)
	Multiple site	8 (9.87)
Smoking status	not Smoker	17 (20.98)
	smoker	52 (64.19)
Drinking status	never-Drinker	29 (35.80)
	Drinker	35 (43.20)
Tumor size (T)	1-2	32 (39.50)
	3-4	46 (56.79)
Regional lymph nodes (N)	No	40 (49.38)
	Yes	38 (46.91)
Clinical Stage	I-II	22 (27.16)
	III-IV	58 (71.60)
Treatment - Radiotherapy (RT)	No	19 (23.45)
	Yes	52 (64.19)
Treatment - Chemotherapy (CT)	No	33 (40.74)
	Yes	34 (41.97)
Surgery + RT or CT	No	48 (59.25)
	Yes	24 (29.62)
Outcome	Dead	45 (55.55)
	Alive	36 (44.44)

**Table 2:** Analysis of the relationship between histological classifications/gradations and neural invasion with prognostic variables (T, nodal metastasis (N), and clinical stage).

Histopathological classification			N (%)	T	Nodal metastasis	Clinical Stage
				*P-value*	*P-value*	*P-value*
Broders' classification	WHO (2005)	Well-differentiated	43 (54.22)	0.77	0.07	0.14
		Moderately and poorly differentiated	36 (43.37)			
Bryne et al.	Malignancy Grading of the Deep Invasive Margins (1992)	≤ 8 points	28 (33.73)	0.11	0.13	0.06
		≥9 points	53 (63.86)			
Brandwein-Gensler et al.	Histologic Risk Assessment (2005)	≤2 points	29 (34.94)	≤0.001 (0.30)*	0.05 (0.22)*	≤0.001 (0.34)*
		≥3 points	52 (62.63)			
	Neural Invasion - S100	No	40 (50.63)	0.31	0.73	≤0.006 (0.31)*
		Yes	39 (49.36)			
	Neural invasion - Foci	Unifocal	9 (23.07)	0.44	0.70	0.01 (0.42)*
		Multifocal	30 (76.92)			
	Neural invasion - Size	<1mm	7 (17.94)	0.52	0.88	0.52
		>1mm	32 (82.05)			

* Cramer's V.

**Table 3:** Univariate and multivariate analyses of survival in relation to clinicopathologic factors.

Variables		Univariate analysis	*P-value*	Multivariate analysis	*P-value*
		HR (95% CI)		HR (95% CI)	
Age	≤50 years	1.12 (0.54-2.32)	0.761	-	-
	≥50 years				
Sex	Male	0.76 (0.39-1.49)	0.425	-	-
	Female				
Smoking status	Not Smoker	1.55 (0.78-3.07)	0.211	-	-
	Smoker				
Drinking status	Never-drinker	1.35 (0.71-2.57)	0.365	-	-
	Drinker				
Tumor (T)	1-2	1.49 (0.81-2.73)	0.197	-	-
	3-4				
Regional lymph nodes (N)	No	0.37 (0.20-0.69)	0.002	2.45 (1.89-5.97)	<0.001
	Yes				
Clinical Stage	I + II	2.60 1.20-5.59)	0.015	4.07 (1.21-13.63)	<0.001
	III + IV				
Radiotherapy	Yes	1.92 (0.99-3.73)	0.054	0.00 (0.00-0.01)	-
	No				
Chemotherapy	Yes	0.98 (0.52-1.84)	0.941	-	-
	No				
Surgery with CT or RT	Yes	2.41 (1.18-4.93)	0.016	2.72 (1.04-7.29)	<0.001
	No				
WHO - Broders (2005)	Well-differentiated	1.70 (0.96-3.02)	0.071	1.00 (0.39-2.59)	0.996
	Moderately/poorly differenciated				
Bryne et al., 1992 Malignancy Grading of the Deep Invasive Margins (1992)	≤8 points	1.98 (1.01-3.90)	0.047	1.00 (0.10-10.0)	1.0
	≥9 points				
Brandwein-Gensler et al., 2005 Histologic Risk Assessment	0-2 points	3.26 (1.61-6.59)	0.001	16.36 (0.16-12.61)	0.761
	≥3 points				
Neural invasion - Foci	Unifocal	3.38 (1.16-9.83)	0.025	4.20 (1.29-13.66)	0.017
	Multifocal				
